# Supplementary material for: Dopamine neuron glutamate cotransmission evokes a delayed excitation in lateral dorsal striatal cholinergic interneurons
Source: eLife. 2018 Oct 8;7:e39786. doi: 10.7554/eLife.39786 (PMC6175576; doi:10.7554/eLife.39786)
Supplement: Figure 3—source data 1. [file elife-39786-fig3-data1.docx]

**Figure 3 – source data** 1

**Statistics for Figure 3B**

Two-way ANOVA, region-cell type comparison

mdStr ChI: n = 20 cells, ldStr ChI: n = 21 cells, mdStr SPN: n = 21 cells, ldStr SPN: n = 20 cells

|  | region | cell type | region*cell type |
| --- | --- | --- | --- |
| df | 1 | 1 | 1 |
| F value | 25.99 | 32.85 | 27.25 |
| p value | 0.000 | 0.000 | 0.000 |

Post hoc: un-paired t-test

| comparison | md-ChI | md-ChI | md-ChI | ld-ChI | ld-ChI | md-SPN |
| --- | --- | --- | --- | --- | --- | --- |
|  | ld-ChI | md-SPN | ld-SPN | md-SPN | ld-SPN | ld-SPN |
| df | 20.45‡ | 27.56‡ | 22.81‡ | 20.11‡ | 20.05‡ | 39 |
| p value | 0.000 | 0.039 | 0.009 | 0.000 | 0.000 | 0.32 |

‡: non-equal variance assumption
