# Supplementary material for: Dopamine neuron glutamate cotransmission evokes a delayed excitation in lateral dorsal striatal cholinergic interneurons
Source: eLife. 2018 Oct 8;7:e39786. doi: 10.7554/eLife.39786 (PMC6175576; doi:10.7554/eLife.39786)
Supplement: Figure 5—source data 1. [file elife-39786-fig5-data1.docx]

**Figure 5 – source data 1**

| **Raw data for Fig 5A**  **GDPβS, charge transfer** | | |  |  |  |
| --- | --- | --- | --- | --- | --- |
| **Ctrl (GTP)** | **single stimulation** | | | **% 0-1 min** | |
| **cell #** | **0-1 min** | **5-6 min** | **>10 min** | **5-6 min** | **>10 min** |
| 1 | 12.8359 | 16.0867 | 13.5416 | 125.3258 | 105.4979 |
| 2 | 29.9549 | 19.3791 | 16.0228 | 64.6943 | 53.4897 |
| 3 | 67.4677 | 44.6694 | 29.9578 | 66.2086 | 44.4032 |
| 4 | 29.2098 | 54.3015 | 35.1104 | 185.9016 | 120.2008 |
| 5 | 19.6048 | 21.1554 | 20.5010 | 107.9093 | 104.5713 |
| 6 | 11.5120 | 13.0312 | 9.6375 | 113.1967 | 83.7167 |
| 7 | 15.5857 | 22.3588 | 26.0079 | 143.4571 | 166.8703 |
| **average** | **26.5958** | **27.2832** | **21.5399** | **115.2419** | **96.9643** |
| SEM | 7.3607 | 5.9462 | 3.4920 | 16.1156 | 15.7251 |
|  |  |  |  |  |  |
| **GDPβS** | **single stimulation** | | | **% 0-1 min** | |
| **cell #** | **0-1 min** | **5-6 min** | **>10 min** | **5-6 min** | **>10 min** |
| 1 | 19.8180 | 4.9473 | 0.1866 | 24.9637 | 0.9414 |
| 2 | 14.7231 | 0.0542 | 0.4263 | 0.3680 | 2.8956 |
| 3 | 9.7088 | 0.2706 | 2.2420 | 2.7867 | 23.0928 |
| 4 | 20.8149 | 0.0064 | 0.3563 | 0.0306 | 1.7117 |
| 5 | 17.0790 | 9.8617 | 0.6736 | 57.7417 | 3.9439 |
| 6 | 21.3552 | 0.9024 | 0.6243 | 4.2258 | 2.9232 |
| 7 | 19.1421 | 1.1498 | 1.2130 | 6.0068 | 6.3369 |
| **average** | **17.5202** | **2.4561** | **0.8174** | **13.7319** | **5.9779** |
| SEM | 1.5623 | 1.3949 | 0.2676 | 8.0166 | 2.9260 |

| **raw data for Fig 5C** | |  |  |  |  |  |  |
| --- | --- | --- | --- | --- | --- | --- | --- |
| **Ctrl** | **Charge** | | | **% pre-drug** | |  |  |
| **cell #** | **Pre drug** | **10 min** | **20 min** | **10 min** | **20 min** |  |  |
| 1 | 16.4641 | 14.0349 | 15.1069 | 85.2455 | 91.7566 |  |  |
| 2 | 30.3495 | 32.9355 | 23.4933 | 108.521 | 77.4092 |  |  |
| 3 | 53.5622 | 60.2482 | 62.4987 | 112.483 | 116.684 |  |  |
| 4 | 11.6825 | 10.0339 | 11.2363 | 85.8883 | 96.1806 |  |  |
| 5 | 45.5072 | 45.2154 | 47.7435 | 99.3588 | 104.914 |  |  |
| 6 | 13.6786 | 17.7545 | 13.8173 | 129.798 | 101.014 |  |  |
| 7 | 55.6709 | 56.8402 | 43.0538 | 102.1 | 77.3363 |  |  |
| **average** | **32.41643** | **33.86609** | **30.99283** | **103.342** | **95.0422** |  |  |
| SEM | 7.235878 | 7.829023 | 7.579102 | 5.89551 | 5.42923 |  |  |
|  |  |  |  |  |  |  |  |
| **D1 antagonist** | **Charge** | | | | **% pre-drug** | | |
| **cell #** | **Pre drug** | **10 min** | **20 min** | **wash** | **10 min** | **20 min** | **wash** |
| 1 | 27.0363 | 26.1843 | 15.1929 | 26.6011 | 96.8487 | 56.1944 | 98.3903 |
| 2 | 57.0111 | 47.0654 | 41.0992 | 47.1215 | 82.5548 | 72.0898 | 82.6532 |
| 3 | 44.9249 | 27.538 | 21.6009 | 43.6382 | 61.2979 | 48.0822 | 97.1359 |
| 4 | 98.1803 | 45.4628 | 28.6364 | 82.468 | 46.3054 | 29.1672 | 83.9965 |
| 5 | 33.8714 | 34.035 | 31.3246 | 37.2328 | 100.483 | 92.481 | 109.924 |
| 6 | 37.3512 | 22.4906 | 14.4741 | 34.4212 | 60.2139 | 38.7514 | 92.1555 |
| 7 | 18.0288 | 18.6169 | 16.7428 | 18.8078 | 103.262 | 92.867 | 104.321 |
| **average** | **45.20057** | **31.62757** | **24.15299** | **41.4701** | **78.7094** | **61.3761** | **95.5109** |
| SEM | 10.0055 | 4.182308 | 3.761 | 7.74562 | 8.6134 | 9.54543 | 3.79676 |
|  |  |  |  |  |  |  |  |
| **mGluR1/5 antagonists** | **Charge** | | | | **% pre-drug** | | |
| **cell #** | **Pre drug** | **10 min** | **20 min** | **wash** | **10 min** | **20 min** | **wash** |
| 1 | 25.8482 | 15.9061 | 10.8624 | 13.838 | 61.5366 | 42.0238 | 53.5356 |
| 2 | 102.277 | 39.1717 | 24.6505 | 22.1319 | 38.2996 | 24.1017 | 21.6392 |
| 3 | 37.1761 | 12.8703 | 13.7394 |  | 34.6198 | 36.9576 |  |
| 4 | 66.8398 | 44.1554 | 37.0995 | 42.3283 | 66.0615 | 55.5051 | 63.328 |
| 5 | 19.1433 | 7.95475 | 5.26794 | 3.89104 | 41.5537 | 27.5185 | 20.3259 |
| 6 | 30.2238 | 20.2191 | 14.8722 | 27.1635 | 66.8979 | 49.2069 | 89.8745 |
| 7 | 36.5336 | 15.4237 | 6.45382 | 6.71495 | 42.2178 | 17.6654 | 18.3802 |
| **average** | **45.43454** | **22.24301** | **16.13511** | **19.3446** | **50.1696** | **36.1399** | **44.5139** |
| SEM | 11.06571 | 5.231523 | 4.250983 | 5.8475 | 5.30444 | 5.21354 | 11.9502 |
|  |  |  |  |  |  |  |  |
| **mGluR1/5+D1 antagonists** | **Charge** | | | | **% pre-drug** | | |
| **cell #** | **Pre drug** | **10 min** | **20 min** | **wash** | **10 min** | **20 min** | **wash** |
| 1 | 55.1481 | 17.2927 | 2.97217 | 16.6446 | 31.3568 | 5.38943 | 30.1816 |
| 2 | 147.225 | 51.2967 | 11.8431 |  | 34.8424 | 8.04422 |  |
| 3 | 14.7742 | 6.61106 | 1.31219 | 6.97531 | 44.7473 | 8.88163 | 47.2128 |
| 4 | 52.3331 | 11.4141 | 8.39222 | 22.4815 | 21.8105 | 16.0362 | 42.9585 |
| 5 | 51.2866 | 15.8586 | 6.95432 | 26.2275 | 30.9215 | 13.5597 | 51.1391 |
| 6 | 61.9905 | 37.2738 | 9.12317 | 24.326 | 60.1282 | 14.717 | 39.2415 |
| 7 | 44.8667 | 5.507 | 1.96127 | 6.56423 | 12.2741 | 4.37133 | 14.6305 |
| **average** | **61.08917** | **20.75057** | **6.079777** | **17.2032** | **33.7258** | **10.1428** | **37.5607** |
| SEM | 15.45847 | 6.476024 | 1.527054 | 3.55032 | 5.84611 | 1.75411 | 5.44592 |
