# Supplementary material for: Dopamine neuron glutamate cotransmission evokes a delayed excitation in lateral dorsal striatal cholinergic interneurons
Source: eLife. 2018 Oct 8;7:e39786. doi: 10.7554/eLife.39786 (PMC6175576; doi:10.7554/eLife.39786)
Supplement: Figure 5—source data 2. [file elife-39786-fig5-data2.docx]

**Figure 5 – source data 2**

**Statistics for Figure 5C**

Mixed ANOVA, time*treatment

Ctrl: n = 7 cells, D1 antagonist: n = 7 cells, mGluR1/5 antagonists: n = 7 cells, D1+mGluR1/5 antagomists: n = 7 cells

|  | time*treatment |
| --- | --- |
| df | 6 |
| F value | 21.38 |
| p value | 0.000 |

Post hoc test within subject: one-way repeated measures ANOVA

|  | Ctrl | D1 | mGluR1/5 | D1+mGluR1/5 |
| --- | --- | --- | --- | --- |
| df | 2 | 2 | 2 | 1.138# |
| F value | 0.91 | 12.24 | 102.93 | 194.66 |
| p value | 0.43 | 0.001 | 0.000 | 0.000 |

#: Greenhouse-Geisser correction for sphericity

Post hoc test between subject: one-way ANOVA, each time point

Main effect

|  | 10 min | 20 min |
| --- | --- | --- |
| df | 3 | 3 |
| F value | 22.17 | 34.80 |
| p value | 0.000 | 0.000 |

Scheffe’s test

| 10 min  comparison | Ctrl | Ctrl | Ctrl | D1+mGluR1/5 | D1+mGluR1/5 | mGluR1/5 |
| --- | --- | --- | --- | --- | --- | --- |
|  | D1 | mGluR1/5 | D1+mGluR1/5 | mGluR1/5 | D1 | D1 |
| p value | 0.096 | 0.000 | 0.000 | 0.388 | 0.001 | 0.043 |

| 20 min  comparison | Ctrl | Ctrl | Ctrl | D1+mGluR1/5 | D1+mGluR1/5 | mGluR1/5 |
| --- | --- | --- | --- | --- | --- | --- |
|  | D1 | mGluR1/5 | D1+mGluR1/5 | mGluR1/5 | D1 | D1 |
| p value | 0.008 | 0.000 | 0.000 | 0.051 | 0.002 | 0.061 |

**Wash of drugs (only washed cells were tested)**

One-sample t-test, to 100% (pre-drug)

D1 antagonist: n = 7 cells, mGluR1/5 antagonists: n = 6 cells, D1+mGluR1/5 antagonists n = 5 cells

|  | D1 | mGluR1/5 | D1+mGluR1/5 |
| --- | --- | --- | --- |
| df | 6 | 5 | 5 |
| p value | 0.28 | 0.006 | 0.000 |

**Statistics for Figure 5D**

One-way ANOVA, at 20 min application

Ctrl: n = 7 cells (same as above), mGluR1 antagonist: n = 7 cells, mGluR5 antagonist: n = 7 cells, mGluR1/5 agonists (same as above): n = 7 cells

|  | time*treatment |
| --- | --- |
| df | 3 |
| F value | 35.30 |
| P value | 0.000 |

Scheffe’s post hoc test

| comparison | Ctrl | Ctrl | Ctrl | mGluR1/5 | mGluR1/5 | mGluR1 |
| --- | --- | --- | --- | --- | --- | --- |
|  | mGluR1/5 | mGluR1 | mGluR5 | mGluR1 | mGluR5 | mGluR5 |
| p value | 0.000 | 0.000 | 0.94 | 0.99 | 0.000 | 0.000 |
