# Supplementary material for: Dopamine neuron glutamate cotransmission evokes a delayed excitation in lateral dorsal striatal cholinergic interneurons
Source: eLife. 2018 Oct 8;7:e39786. doi: 10.7554/eLife.39786 (PMC6175576; doi:10.7554/eLife.39786)
Supplement: Figure 10—source data 1. [file elife-39786-fig10-data1.docx]

**Figure 10 - table supplement 1**

**Statistics for Figure 10A**

One-sample t-test to 1 (1 = no difference between input and IP)

n = 5 replicates (each replicate was from 3 mice), df = 4 for all

| Gene | ChAT | VAChT | D5 | D1 | D2 | mGluR1 | mGluR5 | TrpC3 | TrpC7 |
| --- | --- | --- | --- | --- | --- | --- | --- | --- | --- |
| p value | 0.008 | 0.001 | 0.000 | 0.000 | 0.33 | 0.019 | 0.000 | 0.001 | 0.013 |

**Statistics for Figure 10B**

General linear model multivariate analysis, IP fraction, mdStr/ldStr comparison

n = 5 replicates (each replicate was from 3 mice), df = 1 for all

| Gene | ChAT | VAChT | D5 | D1 | D2 | mGluR1 | mGluR5 | TrpC3 | TrpC7 |
| --- | --- | --- | --- | --- | --- | --- | --- | --- | --- |
| F value | 0.65 | 0.094 | 0.004 | 6.1 | 0.42 | 1.8 | 25.3 | 5.9 | 0.51 |
| p value | 0.44 | 0.77 | 0.95 | 0.039 | 0.53 | 0.22 | 0.001 | 0.042 | 0.50 |
